# Ischemic perconditioning on mesenteric ischemia/reperfusion injury in rats

**DOI:** 10.1590/ACB360903

**Published:** 2021-11-08

**Authors:** Lainy Carollyne da Costa Cavalcante, Giannini Medeiros Rodrigues, Rubens Fernando Gonçalves Ribeiro, Andrew Moraes Monteiro, Ananda Vitória Barros Suzuki Damasceno, Rodrigo Paracampo Couteiro, Edson Yuzur Yasojima, Marcus Vinicius Henriques Brito, Sandro Percário

**Affiliations:** 1 MD. Centro Universitário do Estado do Pará (CESUPA) – Belem (PA), Brazil.; 2 Fellow Master degree. Postgraduate Program in Surgery and Experimental Research – Universidade do Estado do Pará (UEPA) – Belem-PA, Brazil; 3 MD. Universidade do Estado do Pará (UEPA) – Belem (PA), Brazil.; 4 PhD, Full Professor, Head. Division Surgical Abilities - Universidade do Estado do Pará (UEPA) – Belem (PA), Brazil.; 5 PhD, Full Professor. Universidade Federal do Pará (UFPA) – Belem (PA), Brazil.

**Keywords:** Mesenteric Ischemia, Reperfusion, Mesenteric Vascular Occlusion, Oxidative Stress, Rats

## Abstract

**Purpose:**

To evaluate if the perconditioning affects the antioxidant capacity in mesenteric ischemia and reperfusion injury.

**Methods:**

Twenty-one Wistar rats were assigned into three groups, as follows: Sham, IR and rPER. The animals were subjected to mesenteric ischemia for 30 min. rPER consisted of three cycles of 5-min hindlimb ischemia followed by 5 min hindlimb perfusion at the same time to mesenteric ischemic period. After 5 minutes, blood and 5 cm of terminal ileum were harvested for thiobarbituric acid reactive substances (TBARS) and Trolox equivalent antioxidant capacity (TEAC) measurement.

**Results:**

rPER technique was able to reduce intestinal tissue TBARS levels (p<0.0001), but no statistic difference was observed in blood levels between groups, although it was verified similar results in rPER and Sham group. rPER technique also enhanced TEAC levels in both blood (p = 0.0314) and intestinal tissue (p = 0.0139), compared to IR group.

**Conclusions:**

rPER appears as the most promising technique to avoid IR injury. This technique reduced TBARS levels in blood and intestinal tissue and promoted the maintenance of antioxidant defense in mesenteric acute injury.

## Introduction

The blood flow interruption of organs and tissues and their subsequent restoration causes a cascade of molecular events known as ischemia and reperfusion syndrome (IR), perceived in polytrauma and organ transplants^1^, triggering the formation of reactive oxygen species (ROS) immune activation and endothelial dysfunction^2^.

Even though most of the research focus on myocardial infarction, the importance of IR syndrome covers any organ that has a compromised blood supply and subsequently restored^3^, especially the intestine, an organ that is very sensitive to IR^4^.

The intestinal ischemia process presents a diagnostic challenge, because its clinical presentation often occurs in a non-specific way, in addition to the difficulty in recognizing the conditions in which the patient is, before intestinal necrosis occurs. The incidence of intestinal ischemia is increasing, and the mortality rates observed in hospital environments have remained high over the past few decades, ranging from 60 to 80%^5^.

This fact is related to the priority of perfusion to other organs in response to circulatory shock. Even after volume restoration, vasoconstriction persists at all levels of intestinal microvasculature, due to the effects of multiple agents, such as vasoactive substances, and factors derived from the endothelium^6^.

Therefore, several experimental treatment methods have been studied and applied in animal models in order to mitigate the tissue damage caused by intestinal IR. Among these methods, ischemic perconditioning seems to be the most promising strategy for reducing reperfusion injury after ischemia, increasing the tolerance of the intestine against the damage caused by ischemia and reperfusion syndrome^7^^,^ 8.

Schmidt *et al*.^9^ reported the method of remote ischemic conditioning (rPER), which consists on the application of remote ischemic conditioning, by means of a tourniquet performed on the hind limb of pigs, during the main ischemic time, which was effective in preventing reperfusion injury in myocardial ischemia. This protective effect was corroborated by further studies involving myocardial ischemia, and the technique has been expanded to cerebral ischemia^10^^-^ 12.

Thus, the aim of this study was to evaluate if the perconditioning affects the antioxidant capacity in mesenteric ischemia and reperfusion injury.

## Methods

All experiments were performed in accordance with the Brazilian law for scientific use of animals (Law No. 11.794/08) and the National Institutes of Health (NIH) guide for the care and use of laboratory animals (NIH Publications No. 8,023, revised 1978). The research was approved by Animal Use and Care Committee of Universidade do Estado do Pará (UEPA) (Protocol 01/2016).

Twenty-one (12 – 15 wk) male Wistar rats, weighing 290 ± 19 g, were used in this study. The animals were kept in a vivarium of the Experimental Surgery Laboratory, UEPA, with a controlled temperature, light, humidity, and noise. Water and food were provided ad libitum.

### Experimental protocol

**Figure 1:**
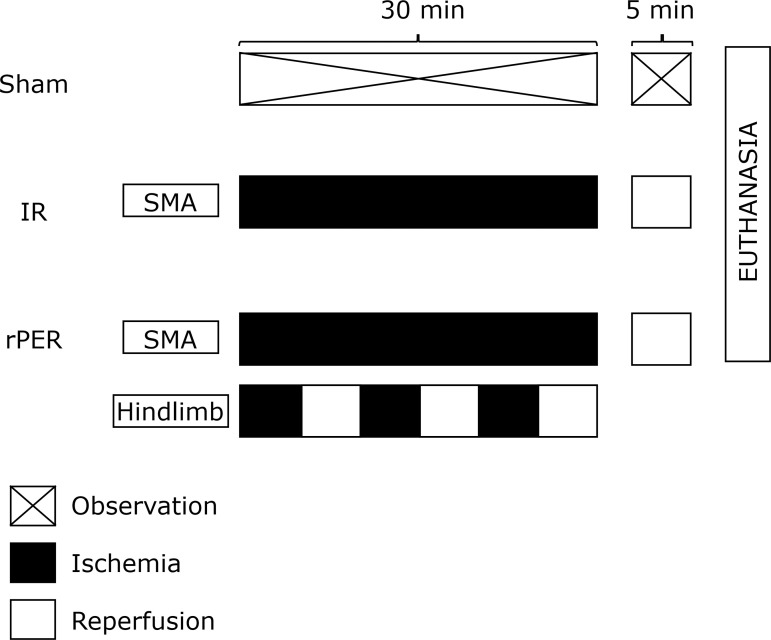
Experimental design. Sham group was not submitted to ischemic conditioning.

IR: ischemia and reperfusion; rPER: remote preconditioning; SMA: superior mesenteric artery.

The animals were randomly assigned into the following three groups (n = 7 for each group):

- Sham group (Sham): the same surgical procedure as in the remaining groups was performed, but no mesenteric ischemia was induced;
- Ischemia and reperfusion group (IR): mesenteric ischemia was induced for 30 min, followed by reperfusion without any form of conditioning;
- Remote perconditioning group (rPER): mesenteric ischemia was simultaneously followed by remote ischemic perconditioning. rPER consisted of three cycles of 5-min left hindlimb ischemia followed by 5-min left hindlimb reperfusion (Fig. 1). Hindlimb ischemia was achieved using an elastic rubber band tied around the thigh of the left leg^8^^,^ 13. The remote ischemic conditioning technique was completed at the same time as the end of the main ischemia^13^^-^ 15, with subsequent reperfusion of 5 minutes.

### Surgical procedures

All surgical procedures were performed in anesthesia (ketamine hydrochloride and xylazine hydrochloride 60 and 6 mg/kg, respectively, i.p.). Through a median longitudinal laparotomy, superior mesenteric artery was occluded by microsurgical clamp application, leading to mesenteric ischemia.

After the ischemia and conditioning protocols, animals were submitted to painless death induced after 5 min of mesenteric reperfusion. Then, blood sample was obtained via puncture of superior mesenteric vein, and 5 cm of terminal ileum was harvested for biochemical analysis. Subsequently, the animals were euthanized by lethal anesthetic doses.

### Laboratory parameters

The samples were homogenized and then immediately centrifuged at 3,000 rpm for 10 min. After centrifugation, samples were directly transferred to Eppendorf tubes and stored at -80°C until assayed. Thiobarbituric acid (TBARS) and the Trolox equivalent antioxidant capacity (TEAC) levels were determined. Biochemical parameters were assessed at the Laboratory for Research on Oxidative Stress, at Universidade Federal do Pará.

### Statistics

The software BioEstat^©^ 5.0 was used. All data were expressed as means ± standard deviation. Analysis of variance, followed by Tukey post hoc test correction, was performed. Statistical significance was assumed at p < 0.05.

## Results

**Figure 2:**
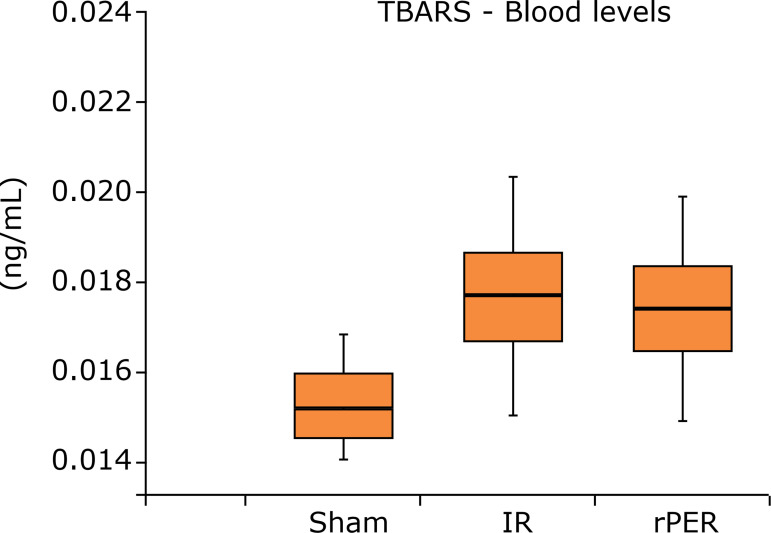
TBARS blood levels (ng/mL) according to groups. Analysis of variance (Tukey post hoc test).

TBARS: Thiobarbituric acid; IR: ischemia and reperfusion; rPER: remote preconditioning.

No animal died during the anesthesia, procedures, or reperfusion period. The ischemic perconditioning technique reduced levels of TBARS in blood sample. IR group (0.018 ± 0.003) had the highest TBARS serum levels in relation to other groups, while Sham group (0.015 ± 0.002) and rPER group (0.017 ± 0.003) presented similar results and had no difference among them (p = 0.1917), even presenting a lower value in relation to IR group (Fig. 2).

**Figure 3:**
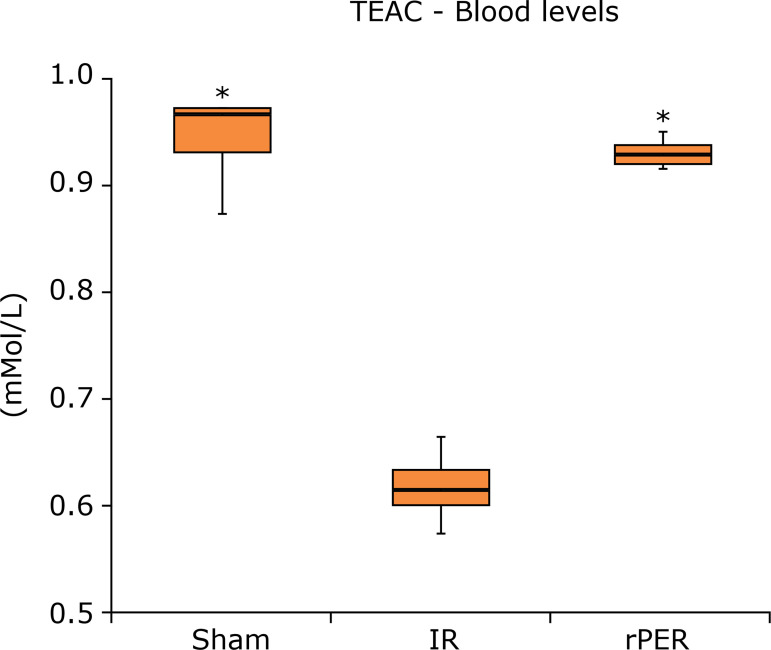
TEAC blood levels (mMol/L) according to groups.

TEAC: Trolox equivalent antioxidant capacity; IR: ischemia and reperfusion; rPER: remote preconditioning; *p<0.05 *vs.* IR. Analysis of variance (Tukey post hoc test).

rPER caused an increase in TEAC serum values (Fig. 3). IR group (0.616 ± 0.033) had the lowest TEAC values compared to other groups, while rPER (0.929 ± 0.014) obtained values close to Sham (0.942 ± 0.043). There was a statistically significant difference between the Sham (p<0.0001) and the rPER (p<0.0001) groups in relation to the IR one.

**Figure 4:**
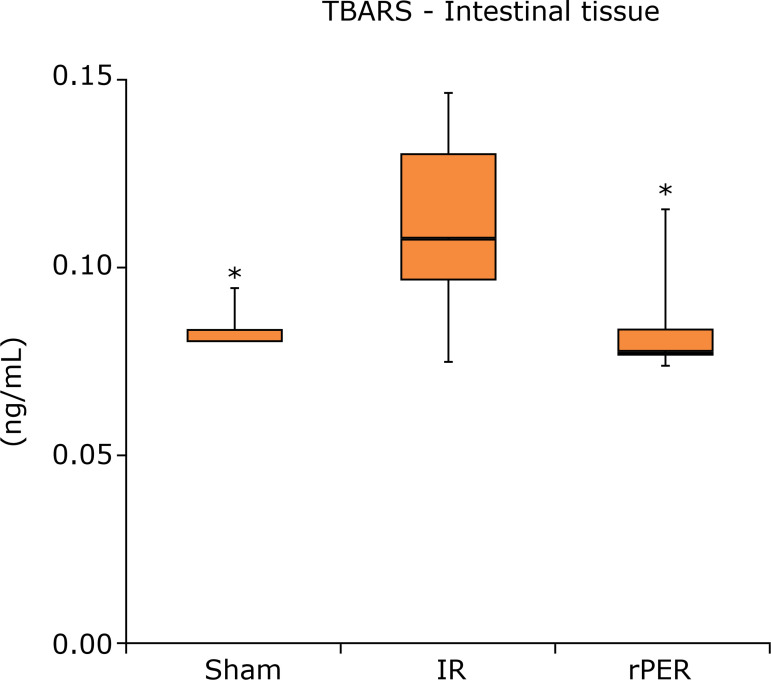
TBARS intestinal tissue levels (ng/mL) according to groups.

TBARS: Thiobarbituric acid; IR: ischemia and reperfusion; rPER: remote preconditioning; *p<0.05 *vs.* IR. Analysis of variance (Tukey post hoc test).

**Figure 5:**
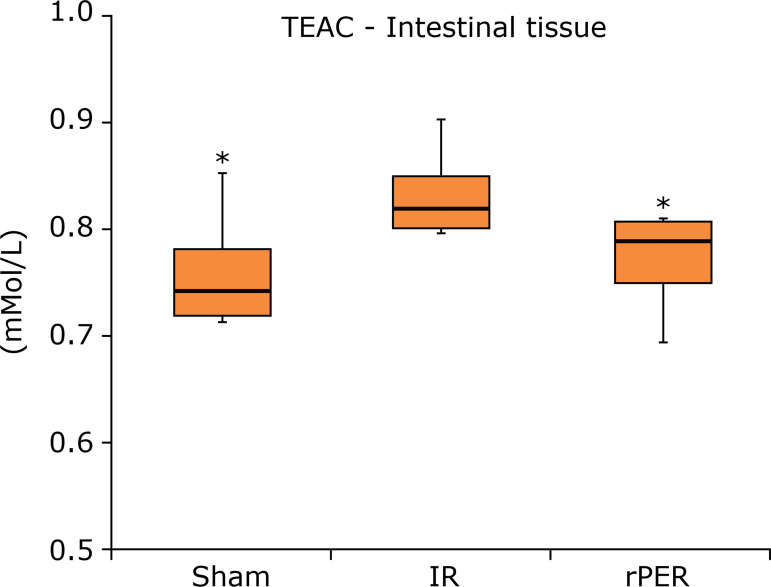
TEAC intestinal tissue levels (mMol/L) according to groups.

TEAC: Trolox equivalent antioxidant capacity; IR: ischemia and reperfusion; rPER: remote preconditioning; *p<0.05 *vs.* IR. Analysis of variance (Tukey post hoc test).

In intestinal tissue, there was a statistically significant difference in TBARS levels (Fig. 4) between Sham (p = 0.0226) and rPER (p = 0.0139) groups compared to IR group. TEAC blood levels (Fig. 5) showed the same effect between Sham (p = 0.0228) and rPER (p = 0.0314) groups in relation to IR group.

The data corresponding to the TBARS and TEAC values measured in blood and intestinal tissue are shown in Table 1.

**Table 1:** Blood and intestine TBARS and TEAC values according to the groups.

Group	N	TBARS (ng/mL)		TEAC (mMol/L)	
		Blood	Intestine	Blood	Intestine
Sham	7	0.015 ± 0.002	0.091 ± 0.014 *	0.942 ± 0.043 *	0.526 ± 0.118 *
IR	7	0.018 ± 0.003	0.112 ± 0.027	0.616 ± 0.033	0.668 ± 0.085
rPER	7	0.017 ± 0.003	0.084 ± 0.014 *	0.929 ± 0.014 *	0.546 ± 0.091 *

TBARS: Thiobarbituric acid; TEAC: Trolox equivalent antioxidant capacity; IR: ischemia and reperfusion; rPER: remote preconditioning;

* p<0.05, *vs.* IR group.

## Discussion

The study of IR is very important, as its deleterious effects can aggravate the clinical conditions of patients undergoing complex surgeries (cardiac, vascular, transplantation, strangulated hernias, and neonatal necrotizing enterocolitis), as well as in certain emergency situations, such as trauma, extensive burns, hemorrhagic shock and septic shock^14^^,^ 16 ^,^ 17.

Knowing that even short periods of mesenteric ischemia can damage the intestinal mucosa, strategies have been developed to minimize its deleterious effects, such as pre-conditioning^7^ and post-conditioning^17^^,^ 18.

However, no studies have been found yet using remote ischemic conditioning technique associated to mesenteric IR injury, in order to assess whether this technique is really capable of attenuating IR injuries in the intestine, while perconditioning was already effective in cases of myocardial^14^, cerebral^20^ and renal^13^ ischemia injury.

The data presented showed that ischemic conditioning was able to reduce oxidative stress, approaching the values of the Sham group. Interestingly, there was no statistical difference between the IR and rPER groups, probably due to the short time of reperfusion, not being enough to alter oxidative stress at the systemic level, or the process of free radical formation being extremely acute. Despite that, it can be said that after performing the ischemic conditioning technique, based on previous studies, there was an additional protection promoted by the execution of remote ischemic conditioning^8^^,^ 10 ^,^ 14.

Regarding the TBARS dosage, there were no statistically significant differences between groups in the blood samples. This fact suggests that there was no systemic repercussion of local changes in this model, or that the great variability of behaviors presented individually by the animals studied may have masked this repercussion^21^^,^ 22.

On the other hand, in the samples of intestinal tissue, there was a difference between sham and perconditioning groups when compared to the IR, suggesting that the ischemic-reperfusion process to which the animals in the IR group were submitted resulted in an increase in the levels of free radicals, as well as, consequently, in the involvement of oxidative stress^23^.

The possible activity of antioxidant enzymes increases such as catalase, superoxide dismutase and glutathione-peroxidase in these groups may be associated with reduction in the degree of lesion of mesenteric IR. It may also have been responsible for the protective activity of the conditioning, which promotes a decrease of neutrophil bearing and activation of nitric oxide to the site of inflammation^8^^,^ 13.

To measure the formation of ROS, TEAC was evaluated in the animals’ blood, which showed how strongly the production of these free radicals can consume the endogenous antioxidant reserve. It was noticed that the ischemic conditioning technique was able to avoid the consumption of these antioxidant substances, allowing to affirm that the technique provides greater protection preventing the formation of ROS, since their values were close to those of the sham group^24^, demonstrating the consumption of systemic antioxidant defenses to mitigate the oxidative damage imposed by the ischemia and reperfusion syndrome to animals.

Furthermore, the significant increase in antioxidant capacity observed at tissue level in the IR group can be explained by the fact that it was subjected to the most intense ischemic injury when compared to rPER group, validating that the conditioning technique proved to be effective in the process of decreased tissue damage.

Although many studies show that increasing antioxidant capacity occurs as a protective factor, it can be said that an increase in this parameter, as observed in the IR group, when evaluated in a short reperfusion period, works as an indicator of greater tissue damage acute by the intrinsic mechanisms of ischemia and reperfusion, such as the formation of EROS^21^^,^ 25.

In addition, a similar behavior was observed when comparing the TEAC values between the sham and rPER groups, which allows to suppose that this technique was able to maintain the antioxidant capacity at normal levels, since the sham group was not submitted to any intervention^26^.

Oxidative stress involves the reactive oxygen species-mediated oxidative degradation of the components of cellular membrane phospholipids followed by formation of peroxy radicals and finally lipid peroxides, that are metabolized, via ß-oxidation pathway, to malondialdehyde (MDA)^27^. In the stomach, for instance, remote ischemic conditioning (RIC) stimulus to the heart or liver significantly reduced gastric mucosal injury, improved gastric blood flow, and suppressed plasma proinflammatory cytokine levels in rat model of gastric ischemic injury^28^, having never previously performed a study in the intestine.

These findings confirm the effective participation of oxidative stress in this model of mesenteric ischemia and reperfusion syndrome in rats and that the application of the isolated ischemic perconditioning technique can protect animals from associated oxidative changes.

## Conclusion

The perconditioning technique was effective in reducing the damage caused by ROS from the mesenteric ischemia and reperfusion syndrome, observed by the decrease in blood and tissue levels of TBARS and in the maintenance of antioxidant capacity, assessed by TEAC, at levels close to parameters of normality.
